# Appointment Density, Message Responsiveness, and Patient Satisfaction

**DOI:** 10.1001/jamanetworkopen.2025.24973

**Published:** 2025-08-04

**Authors:** Lisa Rotenstein, Christopher Toretsky, Elaine Khoong, Urmimala Sarkar, Julia Adler-Milstein

**Affiliations:** 1 Division of Clinical Informatics and Digital Transformation, University of California at San Francisco; 2 Division of General Internal Medicine at UCSF Health, University of California at San Francisco; 3 Division of General Internal Medicine at Zuckerberg San Francisco General Hospital, University of California at San Francisco

## Abstract

This cross-sectional study examines the association of message-based responsiveness and appointment density with patient satisfaction.

## Introduction

Clinicians’ responsiveness to patient needs is associated with patient satisfaction, an important dimension of quality of care.^1^ There is an open question about how physician responsiveness to messages sent via the electronic health record (EHR) and appointment density are associated with patient satisfaction.

## Methods

### Sample

Our sample included primary care and nonprimary care physicians providing outpatient care at an academic medical center in Northern California between January 1, 2020, and March 31, 2022. We limited the sample to months in which the physician had at least 3 Press-Ganey (PG) survey responses. This study was approved by the UCSF institutional review board, which authorized a waiver of informed consent. This study followed the STROBE reporting guideline. All analyses were conducted in SAS, version 9.4, with a 2-sided *P* < .05 considered significant.

### Study Measures

From Epic’s Signal database of EHR use,^2^ we used measures of appointment density (number of appointments per day) and response time to patient medical advice request (PMAR) messages, and from PG patient surveys, we used 6 measures of patient satisfaction after an appointment that we hypothesized would be sensitive to physician-patient interactions. Our dataset was constructed at the physician-month level.

### Statistical Analysis

Statistical analysis was conducted from December 2021 to May 2025. We described physicians’ specialty and sex distribution, as well as the sample’s distribution of appointment density, response time to patient messages, and responses to PG measures of patient satisfaction. We used a generalized estimating equation framework with a logit link and independent correlation structure to assess the association between appointment density and response time with the highest satisfaction (score of 5) on each PG measure. Appointment density and response time were coded as ordinal variables with values 1 to 10 representing 10 percentiles of the variable distribution. Models were adjusted for physician gender and specialty category and clustered by physician.

## Results

Our sample included 998 physicians (50.1% women; 14.8% primary care, 54.7% medical specialists, 26.8% surgical specialists, and 3.7% other specialists). Median appointment density was 7.5 appointments per day (IQR, 5.7-10.0 appointments per day). Median response time to patient messages was 1.0 days (IQR, 0.5-2.2 days). Table 1 shows the proportion of respondents reporting the highest satisfaction on each PG measure.

**Table 1.** Summary Statistics for Press-Ganey Question Responses, Appointment Density, and PMAR Response Time

Survey question	Highest satisfaction, No./total No. (%)^a^
Ease of contacting (eg, email, telephone, and web portal) the clinic	1958/14 277 (13.7)
Concern the care provider showed for your questions or worries	6422/14 327 (44.8)
Explanations the care provider gave you about your problem or condition	6309/14 328 (44.0)
Care provider’s discussion of any proposed treatment (eg, options, risks, and benefits)	5919/14 197 (41.7)
Friendliness or courtesy of the care provider	8136/14 309 (56.9)
Likelihood of your recommending this care provider to others	6861/14 300 (48.0)
Appointment density, mean (SD), No. of appointments/d	8.3 (3.8)
PMAR response time, mean (SD), d	2.8 (9.4)

Abbreviation: PMAR, patient medical advice request.

^a^ Highest satisfaction is all scores of 5 on a 1 to 5 scale.

In adjusted analyses, higher daily appointment density was associated with lower odds of the highest PG satisfaction score for all 6 satisfaction domains (Table 2). A 10-percentile increase in appointment density was associated with 23% lower odds of the highest score for ease of contacting the clinic and 18% lower odds of the highest score for the likelihood of recommending this care provider to others. Message response time was associated only with perceived ease of contacting the clinic.

**Table 2.** Multivariable Models Assessing the Association of Appointment Density and PMAR Message Response Time With the Highest Patient Satisfaction Score on 6 Press Ganey Domains

Variable	Adjusted odds ratio (95% CI)					
	Ease of contacting the clinic^a^	Concern the care provider showed for your questions or worries	Explanations the care provider gave you about your problem or condition	Care provider’s discussion of any proposed treatment^b^	Friendliness or courtesy of the care provider	Likelihood of your recommending this care provider to others
Appointments per day (each additional 10 percentile)	0.77 (0.73-0.81)	0.83 (0.81-0.85)	0.85 (0.82-0.87)	0.83 (0.81-0.86)	0.83 (0.81-0.86)	0.82 (0.80-0.85)
PMAR turnaround time (each additional 10 percentile)	0.92 (0.88-0.96)	0.99 (0.96-1.02)	1.01 (0.98-1.03)	0.99 (0.96-1.02)	1.00 (0.97-1.03)	0.99 (0.96-1.02)
Physician sex						
Female	1.16 (0.88-1.54)	1.15 (0.97-1.38)	1.02 (0.86-1.22)	1.15 (0.96-1.38)	1.19 (1.00-1.41)	1.12 (0.93-1.34)
Male	1 [Reference]	1 [Reference]	1 [Reference]	1 [Reference]	1 [Reference]	1 [Reference]
Physician specialty						
Medical specialty	0.82 (0.59-1.15)	0.82 (0.66-1.03)	0.97 (0.78-1.21)	0.96 (0.77-1.20)	0.93 (0.75-1.17)	0.94 (0.75-1.17)
Other specialty	0.95 (0.52-1.72)	1.03 (0.76-1.40)	1.19 (0.86-1.65)	1.23 (0.88-1.71)	0.97 (0.70-1.35)	1.51 (1.05-2.17)
Surgical specialty	0.79 (0.48-1.29)	1.15 (0.88-1.52)	1.33 (1.02-1.73)	1.17 (0.90-1.53)	1.29 (0.98-1.70)	1.38 (1.06-1.80)
Primary care specialty	1 [Reference]	1 [Reference]	1 [Reference]	1 [Reference]	1 [Reference]	1 [Reference]

Abbreviation: PMAR, patient medical advice request.

^a^ For example, email, telephone, and web portal.

^b^ For example, options, risks, and benefits.

## Discussion

Higher daily appointment density was associated with lower patient satisfaction across numerous domains. Consistent with prior literature,^3,4^ these results potentially reflect the value of lower appointment density in enabling effective listening and patient-physician connections. In addition to its association with patient satisfaction, sufficient appointment time has been identified by physicians as facilitating higher-quality, safe care.^5^ Shorter appointment times are associated with inappropriate prescribing,^6^ a marker of lower-quality care. Despite attention to and health system resourcing of asynchronous, EHR-based communication, faster PMAR response times were not associated with patient satisfaction. Our findings can inform leaders seeking to develop appointment templates and staff teams that balance health system constraints and patient satisfaction.

Strengths include this study’s linkage of measures of physician work with those of patient satisfaction across a large sample of ambulatory care physicians. It is limited by its cross-sectional nature, which does not allow us to infer causation or rule out patients choosing physicians with less-dense schedules. It is also limited by lack of adjustment for patient characteristics or physicians’ years of experience; its focus on a single academic medical center, whose physician work patterns and patient population may not be broadly representative; and its spanning the years of the COVID-19 pandemic. Future studies should characterize the mechanisms behind the associations explored.

## Notes

Supplement.
Data Sharing Statement
