# Supplementary material for: Appointment Density, Message Responsiveness, and Patient Satisfaction
Source: JAMA Netw Open. 2025 Aug 4;8(8):e2524973. doi: 10.1001/jamanetworkopen.2025.24973 (PMC12322797; doi:10.1001/jamanetworkopen.2025.24973)
Supplement: Supplement. — Data Sharing Statement [file jamanetwopen-e2524973-s001.pdf]

Data Sharing Statement

Rotenstein. Appointment Density, Message Responsiveness, and Patient Satisfaction. *JAMA Netw Open*. Published August 04, 2025. doi:10.1001/jamanetworkopen.2025.24973

Data
Data available: No
